# Supplementary material for: Altered Dynamic Functional Connectivity of the Frontoparietal Network in Major Depressive Disorder: Evidence From a Large‐Scale Resting‐State fMRI Study
Source: Brain Behav. 2026 Apr 22;16(4):e71339. doi: 10.1002/brb3.71339 (PMC13103543; doi:10.1002/brb3.71339)
Supplement: Supplementary file 1 — Supplementary Tables: brb371339‐sup‐0001‐Tables.docx [file BRB3-16-e71339-s001.docx]

**Supplementary Materials**

**Supplementary Table S1. Site-specific resting-state fMRI acquisition parameters.**

| **Site** | **Scanner** | **Receive coil** | **TR (ms)** | **TE (ms)** | **Flip angle (°)** | **Slice thickness / gap (mm)** | **Slices** | **Time points** | **Voxel size (mm³)** | **FOV (mm²)** | **Reference** |
| --- | --- | --- | --- | --- | --- | --- | --- | --- | --- | --- | --- |
| Site 15 – Zhongda Hospital, Southeast University | Siemens Verio 3.0T | 12-channel | 2000 | 25 | 90 | 4.0 / 0.0 | 36 | 240 | 3.75 × 3.75 × 4.00 | 240 × 240 | Hou et al., 2018 |
| Site 17 – First Affiliated Hospital of Chongqing Medical University | GE Signa 3T | 8-channel | 2000 | 40 | 90 | 4.0 / 0.0 | 33 | 240 | 3.75 × 3.75 × 4.00 | 240 × 240 | Cao et al., 2016 |
| Site 19 – Anhui Medical University | GE Signa 3T | 8-channel | 2000 | 22.5 | 30 | 4.0 / 0.6 | 33 | 240 | 3.44 × 3.44 × 4.60 | 220 × 220 | Wang et al., 2017 |
| Site 20 – Southwest University | Siemens Tim Trio 3T | 12-channel | 2000 | 30 | 90 | 3.0 / 1.0 | 32 | 242 | 3.44 × 3.44 × 4.00 | 220 × 220 | Cheng et al., 2016; Ye et al., 2015 |
| Site 21 – Beijing Anding Hospital | Siemens Tim Trio 3T | 32-channel | 2000 | 30 | 90 | 3.5 / 0.7 | 33 | 240 | 3.12 × 3.12 × 4.20 | 200 × 200 | Zheng et al., 2018; Jing et al., 2013 |
| Site 22 – Second Xiangya Hospital, Central South University | Philips Gyroscan Achieva 3.0T | 32-channel | 2000 | 30 | 90 | 4.0 / 0.0 | 36 | 250 | 1.67 × 1.67 × 4.00 | 240 × 240 | N/A |
| Site 23 – West China Hospital, Sichuan University | Philips Achieva 3.0T TX | 8-channel | 2000 | 30 | 90 | 4.0 / 0.0 | 38 | 240 | 3.75 × 3.75 × 4.00 | 240 × 240 | Yang et al., 2015 |

*Abbreviations: TR = repetition time; TE = echo time; FOV = field of view. Parameters are reported as provided by the REST-meta-MDD consortium site documentation.*

**Supplementary Table S2. Covariate-adjusted between-group comparisons of temporal dFC metrics.**

| **Metric** | **β (Healthy − MDD)** | **95% CI** | **p** | **q (FDR)** | **Sig.** |
| --- | --- | --- | --- | --- | --- |
| Total transitions | 1.88 | [0.73, 3.03] | 0.001 | 0.004 | ** |
| Fractional occupancy (State 2) | -0.03 | [-0.06, -0.00] | 0.038 | 0.038 | * |
| Mean dwell time (State 2) | -3.22 | [-5.76, -0.69] | 0.013 | 0.019 | * |

*Linear models were fit for each temporal metric with Group as the predictor of interest, adjusting for age, sex, and imaging site. β denotes the adjusted mean difference (Healthy − MDD). q-values are Benjamini–Hochberg FDR-corrected across metrics. Significance codes: * q < 0.05, ** q < 0.01, *** q < 0.001.*

**Supplementary Table S3. State-wise connectivity differences in FPN, non-FPN, and DMN (control analyses).**

| **Network** | **State contrast** | **Mean difference** | **95% CI** | **q (FDR) unadj** | **q (FDR) adj** | **Adj Hedges' g** |
| --- | --- | --- | --- | --- | --- | --- |
| FPN | State 2 − State 1 | -0.09 | [-0.11, -0.08] | <1e-16 | <1e-16 | -2.35 |
| FPN | State 2 − State 3 | -0.17 | [-0.19, -0.15] | <1e-16 | <1e-16 | -3.08 |
| FPN | State 1 − State 3 | -0.07 | [-0.09, -0.05] | <1e-16 | 1.39e-12 | -1.37 |
| Non-FPN | State 2 − State 1 | -0.00 | [-0.00, -0.00] | 3.02e-04 | 0.43 | -0.20 |
| Non-FPN | State 2 − State 3 | -0.00 | [-0.00, -0.00] | 2.02e-07 | 0.43 | -0.22 |
| Non-FPN | State 1 − State 3 | -0.00 | [-0.00, 0.00] | 0.176 | 0.96 | 0.01 |
| DMN | State 2 − State 1 | 0.00 | [-0.02, 0.02] | 0.006 | 0.99 | -0.04 |
| DMN | State 2 − State 3 | 0.00 | [-0.02, 0.02] | 9.46e-04 | 0.99 | 0.02 |
| DMN | State 1 − State 3 | 0.00 | [-0.02, 0.02] | 0.578 | 0.99 | 0.02 |

*Mean differences and 95% CIs summarize within-subject state contrasts. q-values are Benjamini–Hochberg FDR-corrected across state contrasts separately for unadjusted and covariate-adjusted models (age, sex, site). Adjusted Hedges’ g summarizes covariate-adjusted standardized differences.*

**Supplementary Table S4. Descriptive statistics for temporal dFC metrics (full sample).**

| **Variable** | **n** | **Mean** | **SD** | **Median** | **IQR** | **Min** | **Max** |
| --- | --- | --- | --- | --- | --- | --- | --- |
| Transitions | 876 | 23.64 | 8.80 | 25 | 12 | 0.00 | 47.00 |
| Between-state transitions | 887 | 23.74 | 8.96 | 25 | 11 | 0.00 | 47.00 |
| Self-transitions | 887 | 184.26 | 8.96 | 183 | 11 | 161.00 | 208.00 |
| Total transitions from sequence | 887 | 208.00 | 0.00 | 208 | 0 | 208.00 | 208.00 |
| Proportion between-state | 887 | 0.11 | 0.04 | 0.12 | 0.05 | 0.00 | 0.23 |
| Proportion self | 887 | 0.89 | 0.04 | 0.88 | 0.05 | 0.77 | 1.00 |
| Switch rate | 887 | 0.11 | 0.04 | 0.12 | 0.05 | 0.00 | 0.23 |
| Occupancy (State 2) | 876 | 0.53 | 0.23 | 0.54 | 0.34 | 0.00 | 1.00 |
| Dwell time (State 2) | 876 | 15.24 | 19.38 | 9.74 | 8.32 | 0.00 | 209.00 |
| Occupancy (State 1) | 876 | 0.23 | 0.18 | 0.19 | 0.25 | 0.00 | 0.90 |
| Dwell time (State 1) | 876 | 5.75 | 3.52 | 5.44 | 3.78 | 0.00 | 26.86 |
| Occupancy (State 3) | 876 | 0.24 | 0.19 | 0.21 | 0.26 | 0.00 | 0.92 |
| Dwell time (State 3) | 876 | 6.58 | 4.59 | 6.00 | 4.37 | 0.00 | 48.25 |

*IQR = interquartile range. Values are reported for participants with available data for each metric.*

**Supplementary Table S5. Unidimensional analysis: mean FPN connectivity strength (collapsed across windows).**

| **Outcome** | **Model** | **n (HC)** | **HC mean ± SD** | **n (MDD)** | **MDD mean ± SD** | **Group effect (MDD − HC)** | **95% CI** | **p** | **Effect size** | **95% CI (effect)** |
| --- | --- | --- | --- | --- | --- | --- | --- | --- | --- | --- |
| FPN mean strength | Welch t-test (unadjusted) | 441 | 0.18 ± 0.06 | 435 | 0.17 ± 0.06 | -0.01 | [-0.02, 0.00] | 0.052 | g = -0.13 | [-0.27, 0.00] |
| FPN mean strength | OLS (adjusted: age, sex, site) |  |  |  |  | -0.01 | [-0.01, 0.00] | 0.072 | stdβ/σ_resid = -0.12 | [-0.26, 0.01] |

*This analysis tests whether the case–control signal is captured by an overall mean-strength difference. Unadjusted effects are from Welch’s t-test; adjusted effects are from OLS controlling for age, sex, and imaging site. CIs correspond to those reported by the analysis output (bootstrap percentile where applicable).*

**Supplementary Table S6. Exploratory clinical stratification analyses (medication status; episode status).**

| **Medication** | **Episode** | **Model** | **Metric** | **n (MDD subgroup)** | **n (HC)** | **β (Subgroup − HC)** | **95% CI** | **p** | **q (FDR)** | **Sig.** |
| --- | --- | --- | --- | --- | --- | --- | --- | --- | --- | --- |
| Yes |  | Unadjusted | Transitions | 139 | 441 | -1.84 | [-3.47, -0.22] | 0.03 | 0.08 | ns |
| Yes |  | Unadjusted | Occupancy (State 2) | 139 | 441 | 0.01 | [-0.03, 0.06] | 0.54 | 0.54 | ns |
| Yes |  | Unadjusted | Dwell time (State 2) | 139 | 441 | 2.15 | [-1.30, 5.60] | 0.22 | 0.33 | ns |
| Yes |  | Adjusted | Transitions | 139 | 441 | -1.36 | [-3.00, 0.28] | 0.10 | 0.31 | ns |
| Yes |  | Adjusted | Occupancy (State 2) | 139 | 441 | 0.00 | [-0.04, 0.04] | 0.97 | 0.98 | ns |
| Yes |  | Adjusted | Dwell time (State 2) | 139 | 441 | 1.68 | [-1.82, 5.18] | 0.35 | 0.52 | ns |
| No |  | Unadjusted | Transitions | 164 | 441 | -2.32 | [-3.86, -0.77] | 0.003 | 0.010 | * |
| No |  | Unadjusted | Occupancy (State 2) | 164 | 441 | 0.05 | [0.01, 0.09] | 0.026 | 0.026 | * |
| No |  | Unadjusted | Dwell time (State 2) | 164 | 441 | 4.74 | [1.15, 8.32] | 0.010 | 0.014 | * |
| No |  | Adjusted | Transitions | 164 | 441 | -2.06 | [-3.61, -0.50] | 0.00 | 0.02 | * |
| No |  | Adjusted | Occupancy (State 2) | 164 | 441 | 0.04 | [-0.00, 0.07] | 0.07 | 0.07 | ns |
| No |  | Adjusted | Dwell time (State 2) | 164 | 441 | 4.44 | [0.80, 8.09] | 0.020 | 0.03 | * |
|  | First | Unadjusted | Transitions | 235 | 441 | -2.24 | [-3.60, -0.87] | 0.001 | 0.004 | ** |
|  | First | Unadjusted | Occupancy (State 2) | 235 | 441 | 0.03 | [-0.00, 0.07] | 0.055 | 0.055 | ns |
|  | First | Unadjusted | Dwell time (State 2) | 235 | 441 | 3.28 | [0.21, 6.34] | 0.036 | 0.054 | ns |
|  | First | Adjusted | Transitions | 235 | 441 | -1.88 | [-3.26, -0.51] | 0.00 | 0.02 | ** |
|  | First | Adjusted | Occupancy (State 2) | 235 | 441 | 0.03 | [-0.01, 0.06] | 0.16 | 0.16 | ns |
|  | First | Adjusted | Dwell time (State 2) | 235 | 441 | 2.87 | [-0.25, 6.00] | 0.070 | 0.11 | ns |
|  | Recurrent | Unadjusted | Transitions | 68 | 441 | -1.63 | [-3.81, 0.55] | 0.142 | 0.213 | ns |
|  | Recurrent | Unadjusted | Occupancy (State 2) | 68 | 441 | 0.02 | [-0.04, 0.07] | 0.551 | 0.551 | ns |
|  | Recurrent | Unadjusted | Dwell time (State 2) | 68 | 441 | 4.50 | [-0.29, 9.29] | 0.066 | 0.197 | ns |
|  | Recurrent | Adjusted | Transitions | 68 | 441 | -1.24 | [-3.41, 0.92] | 0.26 | 0.39 | ns |
|  | Recurrent | Adjusted | Occupancy (State 2) | 68 | 441 | 0.06 | [-0.05, 0.06] | 0.85 | 0.85 | ns |
|  | Recurrent | Adjusted | Dwell time (State 2) | 68 | 441 | 3.92 | [-0.86, 8.71] | 0.10 | 0.32 | ns |

*Subgroup analyses compare each MDD stratum against healthy controls using unadjusted and covariate-adjusted models (age, sex, site). q-values are Benjamini–Hochberg FDR-corrected across metrics within each stratified set. Medication and episode information were available for subsets of patients; sample sizes refer to those with available subgroup labels.*
